# Vascular Endothelial Damage in COPD: Where Are We Now, Where Will We Go?

**DOI:** 10.3390/diagnostics14090950

**Published:** 2024-04-30

**Authors:** Gianluca Screm, Lucrezia Mondini, Francesco Salton, Paola Confalonieri, Liliana Trotta, Mariangela Barbieri, Antonio Romallo, Alessandra Galantino, Michael Hughes, Selene Lerda, Marco Confalonieri, Barbara Ruaro

**Affiliations:** 1 Pulmonology Unit, Department of Medical Surgical and Health Sciences, University of Trieste, Hospital of Cattinara, 34149 Trieste, Italy; 2 Division of Musculoskeletal and Dermatological Sciences, Faculty of Biology, Medicine and Health, The University of Manchester & Salford Royal NHS Foundation Trust, Manchester M6 8HD, UK; 3 Graduate School, University of Milan, 20149 Milan, Italy

**Keywords:** chronic obstructive pulmonary disease (COPD), emphysema, nailfold video capillaroscopy (NVC), endothelial dysfunction, nitric oxide (NO)

## Abstract

Background: Chronic obstructive pulmonary disease (COPD) has higher rates among the general population, so early identification and prevention is the goal. The mechanisms of COPD development have not been completely established, although it has been demonstrated that endothelial dysfunction plays an important role. However, to date, the measurement of endothelial dysfunction is still invasive or not fully established. Nailfold video capillaroscopy (NVC) is a safe, non-invasive diagnostic tool that can be used to easily evaluate the microcirculation and can show any possible endothelial dysfunctions early on. The aim of this review is to evaluate if nailfold microcirculation abnormalities can reflect altered pulmonary vasculature and can predict the risk of cardiovascular comorbidities in COPD patients. Methods: A systematic literature search concerning COPD was performed in electronic databases (PUBMED, UpToDate, Google Scholar, ResearchGate), supplemented with manual research. We searched in these databases for articles published until March 2024. The following search words were searched in the databases in all possible combinations: chronic obstructive pulmonary disease (COPD), endothelial damage, vascular impairment, functional evaluation, capillaroscopy, video capillaroscopy, nailfold video capillaroscopy. Only manuscripts written in English were considered for this review. Papers were included only if they were able to define a relationship between COPD and endothelium dysfunction. Results: The search selected 10 articles, and among these, only three previous reviews were available. Retinal vessel imaging, flow-mediated dilation (FMD), and skin autofluorescence (AF) are reported as the most valuable methods for assessing endothelial dysfunction in COPD patients. Conclusions: It has been assumed that decreased nitric oxide (NO) levels leads to microvascular damage in COPD patients. This finding allows us to assume NVC’s potential effectiveness in COPD patients. However, this potential link is based on assumption; further investigations are needed to confirm this hypothesis.

## 1. Introduction

Chronic obstructive pulmonary disease (COPD) is a global problem and currently is the third cause of death worldwide, leading to almost 6 million deaths annually around the globe [1,2,3,4,5,6]. While the management of this disease is a hugely important objective, at the same time, it is quite complicated and challenging because COPD is commonly exhibited in patients with multiple comorbidities [2,3,4,5,6,7,8]. COPD often occurs with some relevant cardiovascular comorbidities, such as arterial hypertension, heart failure, acute myocardial infarction, stroke, or peripheral arterial disease. These comorbidities largely contribute to increasing COPD’s severity and accelerate the disease’s progression, leading to progressive deterioration in quality of life, resulting in poorer clinical outcomes [3,4,5,6,7,8,9,10]. In-depth studies of this pathology and progress in our knowledge of the underlying pathogenetic mechanisms have led to evidence in the literature that support a strict correlation between chronic systemic inflammation, which characterizes COPD patients, and comorbidities that are commonly associated with COPD, especially cardiovascular comorbidities [11,12,13,14,15,16,17,18,19,20]. In particular, this generally increased inflammatory state plays an important role in promoting atherosclerotic mechanisms, which are obviously a main cause of cardiovascular events [20,21,22,23,24,25,26,27,28,29,30,31,32].

Except for a few unusual situations, COPD can undoubtedly be considered a medical condition that is specifically related to old age. Both prevalence and incidence of this disease are higher within age groups >50 years old, and the average age of patients with COPD is 70 years old [4,5,6,7,8].

COPD is a virtually untreatable disease; it is defined as chronic airflow obstruction, not fully manageable, and with an inevitable progression over time [5,6,7,8,9]. In this chronic lung disease, there is increased inflammation in the airways and pulmonary remodelling, often related to parenchymal destruction and primarily responsible for emphysema; in addition, mucous hyper-secretion and loss of elastic recoil are major events that are related to the development of airflow obstruction [6,7,8,9,10]. These mechanisms underlie the typical symptomatology of COPD, in particular dyspnoea, wheezing, fatigue, cough, and possibly sputum production [6,7,8,9,10].

It is stated that cigarette smoking is the main risk factor of COPD; in fact, the literature has proven that non-smokers develop this disease less frequently [7,8,9,10]. Nevertheless, smoking is not the only cause of this disease, and as a matter of fact, only 30% to 50% of smokers develop COPD, so there are other major risk factors that play an almost equally key role related to the disease [5,8]. For example, exposure to high levels of indoor and outdoor air pollution, tuberculosis infection, frequent childhood infections, or childhood asthma all contribute to COPD development [5,8].

On the other hand, there is also a small percentage of people affected by COPD due to genetic abnormalities leading to a deficiency of alpha-1 antitrypsin, which plays a major role in protecting the lungs against proteolytic damage [5,6,7,8,9].

Diagnosis of COPD should be made starting from an analysis of exposure history, considering both smoking and the other main risk factors previously indicated, then moving towards clinical examination and instrumental exams [2,3,4,5,6].

A firm diagnosis is mainly based on spirometry parameters which show airway obstruction, characterized by irreversibility, revealed after the administration of a bronchodilator.

Moreover, the two major spirometry parameters of interest are FEV1 (forced expiratory volume during the first second) and the FEV1/FVC ratio, both of which are decreased in COPD patients.

So, a positive patient history, clinical signs, and a suggestive pulmonary function test are enough for a firm diagnosis. According to GOLD classification, COPD severity is graded according to the predicted FEV1 value as follows: mild (FEV1 > 80%), moderate (FEV1 from 50% to 80%), severe (FEV1 from 30% to 50%) and very severe (FEV1 < 30%) [2,3,4,5,6,32,33,34,35,36,37,38].

One the most serious challenges in COPD management is to control, treat, and prevent exacerbations of the disease, defined as severe and acute worsening of the symptoms and/or patient wellbeing. Within the Asia–Pacific region, almost 50% of patients with COPD had exacerbations and almost 20% of them needed hospitalization due to severe symptoms [9].

## 2. Role of Endothelium in COPD

COPD is a disorder strictly associated with a general increased inflammation status. Almost all researchers have focused on the role of inflammatory cells in COPD and did not give proper attention to endothelial function [38,39,40,41,42,43,44,45,46,47].

Nowadays, it is well established that, in COPD, the pulmonary vasculature does not work properly due to endothelial dysfunction, which is associated with disease severity and clinical outcomes; in addition, it is probably also an important pathogenic factor [13,14,15].

The vascular endothelium is composed of a monolayer of endothelial cells that lies between the luminal surface of the vasculature and the vessel wall [16]. In the lung, the endothelium represents the interface between the blood and other lung compounds, such as the parenchyma and airways, so it has a critical position and must function properly to ensure the balance of the whole system. Like in the lung, the endothelium has other similar critical roles for the whole body [48,49,50,51,52,53,54,55,56,57,58].

Endothelial activity plays a key role in the development of COPD, as shown by Table 1.

So, in COPD, the endothelium appears to not work as it is supposed to. Endothelial dysfunction is an abnormal action of the endothelium, mainly characterized by decreased vasodilatation, promotion of the inflammatory state, and loss of its anti-thrombotic functions [13,17,18]. There are three main mechanisms responsible for endothelial dysfunction: oxidative stress, which is highly increased in COPD; systemic inflammation, a major characteristic in the pathogenesis of COPD; and in particular the reduced availability of nitric oxide (NO) [13,17,18]. The latter is one of the most important vasodilator molecules, as it inhibits crucial events in the development of atherosclerosis, and abnormalities in its levels reflect mainly in the microcirculation [17]. A link seems to exist between oxidative stress and NO, since reactive oxygen species (ROS) act as enzymatic inhibitors of NO synthase, leading to decreased levels of NO [57]. Loss of NO-dependent vasodilatation may result in a reduced perfusion, as well as in the maintenance of inflammatory status [58].

Patients with severe COPD or who experience exacerbations of the disease demonstrate a greater rate of circulating inflammatory cells, with upregulation of various molecules, among which are cytokines, chemokines, and acute-phase proteins cells [20]. The literature states that some abnormalities within circulating cells may maintain the inflammatory state and contribute to the different mechanisms indicated in Table 1 [18]. Endothelial dysfunction plays an important role in COPD’s severity, mainly contributing to the pathogenesis of atherosclerosis, an important comorbidity in COPD patients that leads to the worst disease outcomes. Furthermore, the degree of endothelial dysfunction seems to have a significant prognostic value in cardiovascular events [18].

Nevertheless, some works in the literature showed evidence of alterations in lung vascularity in patients in the early stages of COPD, suggesting that these alterations may take part in the initial development of the disease, not only as an alteration during more severe COPD stages or during exacerbations [19].

Despite the proven role of the endothelium in the pathogenesis of various diseases and the scientific community’s focus on the endothelium, data on the peripheral changes that occur in COPD, peripheral abnormalities, and pulmonary function tests are still missing.

Endothelial dysfunction is present in both the coronary circulation and in other microvascular networks, such as retinal vessels, which may suggest that pulmonary endothelial dysfunction may have an effect on other organs [10,11,17]. In addition, endothelial dysfunction is not restricted to central circulation, but it also affects smaller vessels in the peripheral microcirculation [21]. Moreover, these findings suggest that microvascular assessment may be used as an alternative marker of pulmonary endothelial dysfunction, allowing us to detect cardiovascular risk factors that can worsen existing COPD. This possible link has been previously shown to be potentially associated with induced retinal vasodilation [11]. Over the last few years, new and non-invasive methods such as nailfold video capillaroscopy (NVC) have gained relevance in the assessment of microvascular activity in unconventional diseases. Up to now, there is a lack of literature regarding these themes, despite the potential of these methods in terms of their diagnostic and prognostic relevance.

## 3. Nailfold Video Capillaroscopy (NVC)

NVC is a non-invasive technique employed to evaluate nailfold microcirculation, allowing us to define characteristics and functionalities of the nailfold capillaries [22]. Nowadays, it plays a well-established role in the assessment of Raynaud’s phenomenon (RP) and systemic sclerosis (SSc); starting from 2013, NVC assessment of scleroderma pattern was included in the classification criteria for SSc.

An initial clinical trial showed that patients who suffer from RP without NVC abnormalities, monitored with a strict NVC follow-up, may show new possible NVC alterations that can predict a transition to secondary Raynaud phenomenon (SRP) [51].

Moreover, the importance of NVC is becoming an increasingly valuable asset for other diseases such as antineutrophil cytoplasmic antibody (ANCA)-associated vasculitis (AAV) and non-rheumatic diseases, even though its role in this field is still not well assessed [23].

NVC is a safe technique that can be used to easily evaluate several microvascular parameters: qualitative assessment (global capillaries array and morphology), semiquantitative analysis (presence of giant capillaries, capillary architecture disorganization, microhaemorrhages, neoangiogenesis, and capillary loss), quantitative analysis (estimates of capillary density, avascular areas, diameter of enlarged capillaries), and dynamic parameters (blood flow velocity) [24,25].

New methods, including laser speckle contrast analysis (LASCA), able to measure peripheral blood perfusion are being tested and are showing good results [49].

Patients with SSc present microvascular changes mainly due to endothelial cell dysfunction, characterized by an imbalance between the decrease in vasodilatation levels and the upregulation of vasoconstrictor molecules [26,27]. This impairment of microvascular homeostasis leads to microvascular abnormalities such as increase in capillary wall permeability and progressive microvascular weakness, conditions that appear in NVC as microhaemorrhages and local oedema [26,27]. In SSc patients, the resulting hypoxia leads to an overproduction of VEGF that determines the presence of bizarre and ramified capillaries, reflecting a neoangiogenic process. Nevertheless, in these patients, the chronic hypoxia induces irreversible microangiopathy, seen in NVC as avascular areas [27].

Several studies have demonstrated NVC’s usefulness in lung diseases such as idiopathic pulmonary fibrosis (IPF) [29]. The underlying mechanism of endothelial dysfunction of SSc patients shares certain characteristics with COPD, despite the different levels of VEGF that are upregulated in SSc patients, in contrast to COPD, where they are underregulated [13,28].

The structural assessment of nailfold microvasculature could add important information allowing clinicians to better establish the cardiovascular risk and perhaps even COPD severity. Despite the proven association between retinal vessel diameter and cardiovascular risk, in-depth studies have not been conducted yet [10]. Studying microvascular reactivity is one of the most effective ways to assess endothelial function, as alterations in reactivity could be used as a surrogate marker of its function, but more data are required to confirm this theory [10,11,12,30].

However, the potential to evaluate the endothelial function of large and small vessels in the periphery with non-invasive methods is promising. Retinal imaging, finger-pulse plethysmography, and especially brachial artery flow-mediated dilation (FMD) have revealed significant results in COPD. The interest for new reliable methods of measuring endothelial functions is growing, even if the prognostic and diagnostic utility is still unclear. There are neither data nor known limitations regarding the possible utility of NVC in COPD, so the goal of this review is to assess any possible use of NVC in COPD patients based on the evidence gained from other diseases.

## 4. Assessment of Endothelial Dysfunction in COPD Patients

The research work retrieved 17 articles, of which 7 were excluded because they lacked data of interest. In detail, three studies assessed the microvasculature through high-resolution images of retinal blood vessels [10,12,31]; three studies evaluated endothelial functioning by both FMD and nitrate-mediated dilatation (NMD) [32,35,36]; one review evaluated articles with different assessment methods such as optical coherence tomography (OCT), retinal fundus imaging, retinal oximetry, and colour Doppler ultrasonography [37]; one study measured peripheral endothelial dysfunction by using the EndoPat-2000 [33]; one study measured retinal vessel calibres, urine albumin, and myocardial blood flow on MRI as markers of microvascular dysfunction [39]; and lastly, one review included several methods for detecting endothelial dysfunction such as FMD, peripheral arterial tonometry (PAT), flow-mediated skin fluorescence (FMSF), and forearm blood flow after bradykinin infusion (VOP) [34].

### 4.1. Article Features

The 10 selected articles are listed in the table below, with the aim of the table being to highlight their similarities and differences and especially to define the aim of each paper and the different methods used to assess possible endothelial dysfunction in COPD patients. The main clinical papers are listened in Table 2.

### 4.2. Association between Microvascular Impairment and COPD

Spirometry was performed to determine the main pulmonary function parameters of interest, and the conclusions are listed in Table 3.

As stated in Table 3, retinal vessel diameters and, more generally, the functional and structural analysis of retinal vasculature are considered good markers of changes in endothelial functionality, as well as in cardiovascular risk assessment, since an association exists between hypertension and systemic inflammation [12,37]. It is known that COPD patients have significantly greater rates of wider retinal vessels compared to healthy controls [39]. Moreover, Harris B et al. described a potential link between retinal vessel diameters and decreased lung function (assessed by FEV1), and they defined a possible association between retinal vascular calibre and brachial artery FMD [39]; these findings were not confirmed by other studies considered in our review [10,12]. FMD is a widely used method aimed to evaluate endothelial function which seems to be correlated with decreased performance in pulmonary function tests and COPD severity [36,39].

COPD patients have a linear relationship between aerobic capacity and disease mortality. In addition, some articles showed that both retinal vessel evaluation and FMD could have a role in the assessment of endothelial response to exercise [10,33]. In particular, Vaes et al. did not detect any significant changes in vessel diameters during exercise in COPD patients, potentially due to an inadequate endothelium-mediated vasodilatory response [10]. On the other hand, endothelial function was shown to be strictly related to aerobic exercise, since it was negatively associated with VO2 values [33]. Overall, a more impaired FMD is correlated with worsening bronchial obstruction in COPD [32,34,36].

Other methods have been evaluated by Vaes et al. in 2020 for the assessment of endothelial dysfunction in COPD patients. Skin AF showed associations with retinal vessel diameters and reductions in the predicted FEV1% and FEV1/FVC. Moreover, this study also suggested that skin AF is also related to cardiovascular risk [31].

There is not enough evidence towards EndoPAT as a useful tool in COPD patients. Nevertheless, while some studies did not detect an adequate association between EndoPAT and FMD [17,33], it is proven to provide a potential value for predicting cardiovascular events and early atherosclerosis [17]. Moreover, this method can easily detect peripheral finger endothelial dysfunction, which is related to metabolic and cardiovascular risk factors, playing a role as a potential tool in predicting non-obstructive coronary atherosclerosis [17].

Asset endothelial function through the evaluation of peripheral fingers could have potential for cardiovascular risk assessment in COPD patients, considering that cardiovascular comorbidities are, aside from exacerbations, the first cause of poorer outcomes in COPD patients. Nailfold video capillaroscopy has never been used in this disease, and no evaluation of the relationship between capillaroscopic alteration and cardiovascular risk, exacerbations risk, or COPD severity has ever been conducted. Nevertheless, NVC is starting to see potential use in chronic pulmonary diseases such as idiopathic lung fibrosis, where the main alterations found were higher rates of neoangiogenesis and lower capillary density [29].

## 5. Potential Role of NVC in COPD

**Figure 1:**
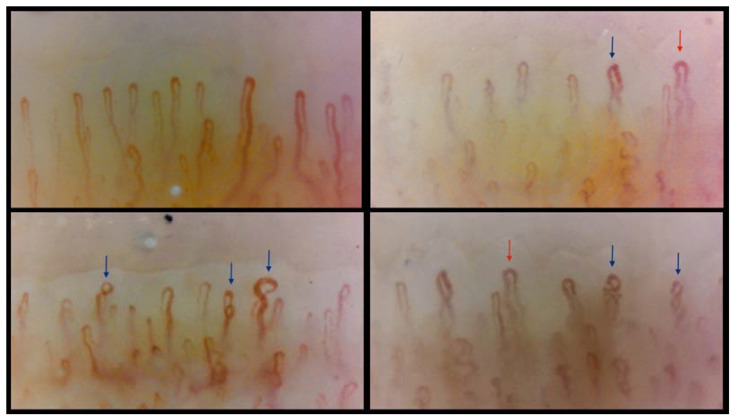
Capillaroscopic images recorded at our centre in a 78-year-old patient with moderate COPD, emphysematous phenotype: capillary density is preserved and capillaries with tortuous and winding morphology (red arrow) and capillaries with multiple and single cross-overs (blue arrow) are associated with normoconformed capillaries.

The link between nailfold microvascular disarrangement, which is seen in NVC (Figure 1), and endothelial dysfunction, primarily assessed via FMD, is yet to be clarified in COPD. Few studies have evaluated this possible relationship [40,41,42,43,44,45].

Peripheral microvascular endothelial dysfunction has shown to play a role in the development of RP, where decreasing levels of NO lead to vasospasms reflected by nailfold microcirculation, suggesting a close relationship between endothelial dysfunction (defined as a decrease in NO availability) and capillaroscopic abnormalities that are usually present in SRP patients [40]. In patients with secondary RP, the reduction in FMD is reported to be associated with nailfold microcirculation impairment. Moreover, an inverse association has been found between FMD and microangiopathy evolution score, corroborating the theory explained before; so, NVC has been proposed has a useful tool for the evaluation of endothelial dysfunction [44]. The same inverse relation was detected by Rollando et al. in 2010 in a study which enrolled SSc patients within asymptomatic cardiovascular disease and found a reduction in FMD values in early NVC microangiopathy patterns and lower FMD values according to late NVC microangiopathy patterns [41]. FMD is a marker for vascular function and NO release, both of which are demonstrated to be significantly reduced in COPD and SSc patients [13,28,41] and which seem to correlate with peripheral microvascular injury [40,41,42,43,45]. FMD, together with NVC, may play an important role in the assessment of vascular damage in SSc patients and may also predict future vascular complications, allowing clinicians to better stratify the cardiovascular risk in those patients [43,45].

Controversially, a possible link between NVC and retinal imaging has not been found yet, despite few works explaining that retinal abnormalities may reflect vascular damage in SSc patients [46,47]. In 2020, Jakhar et al. found that retinal abnormalities were mostly associated with greater NVC alterations. However, no statistically significant data were found, and clinical relevance of this possible relationship must be analysed further to be confirmed [48].

So, taking everything into account, the association between NVC and FMD has been established in patients with SSc and SRP. The main cause of microvascular damage in those patients has been shown to be lack of NO availability, a main marker of endothelial damage in COPD patients [13,28,41]. All of these findings lead to the speculation that NVC may play a role in COPD patients, particularly together with FMD or other methods that assess endothelial damage. Moreover, clinical applications of FMD are difficult to perform due to the strict protocols involved, the expensive equipment, and the need for highly qualified operators and a controlled environment [34]. Nevertheless, one study has shown that FMD provides evidence for the reproducibility of endothelial function assessment in COPD patients [35]. However, this evidence is not supported by other studies and needs further investigations. On the other hand, NVC is nowadays a non-invasive, easily reproducible, non-operator-dependent tool, and thanks to initial evidence of its possible overlap with FMD values, its possible role in evaluating and studying COPD patients should be assessed.

NVC’s enhanced ease-of-use are also due to new advances in digital imaging technology which have led to the development of a validated, fully automated algorithm, AUTOCAPI, with the aim of making NVC more easily reproducible and accurate [50].

We are aware that all these hypotheses strike us more as interpretations and suppositions than as scientifically well-founded evidence. To date, systematic studies characterizing the nailfold microvasculature in patients with COPD are missing, and no relevant articles were found concerning NVC in those patients.

## 6. Conclusions

In conclusion, this review pointed out that the COPD is not just a lung disease, but is strictly connected with systemic involvement. We can therefore consider COPD as a systemic disease. This could seriously open the door for new, possibly effective prospective tools, such as nailfold video capillaroscopy, that aim to evaluate different parameters concerning the microcirculation and can give more meaningful information about systemic endothelial function. This could add important medical evidence to help better understand the role of endothelial dysfunction as a primary factor leading to COPD development and progression, and at the same time, encourage the development of cardiovascular comorbidities and/or COPD exacerbations, which are the main factors of the worst patient outcomes.

## 7. Future Developments

More effective early diagnostic methods are needed for COPD, with the perspective that such tools will lead to early diagnosis and be able to predict the risk of disease exacerbations or cardiovascular comorbidities.

A further area of development is to consider NVC as a possible option available to better stratify COPD patients, since NVC is providing to be a useful tool for many diseases. Endothelial damage is proven to be a significant event during COPD, and therefore, greater understanding of its assessment can reveal novel targets in the management and prevention of this disease.

## Figures and Tables

**Table 1:** Endothelial mechanism in COPD. In the lung, the endothelium has a critical position and must function properly to ensure the balance of the whole system. The endothelial activity plays a key role in the development of the disease.

Transendothelial migration (TEM)	TEM involves the migration of neutrophils through the endothelial cell, bypassing the normal paracellular route which involves its junction [52]. This mechanism appears to be upregulated in patients with COPD, with a unique pathway [13,52]. Since NO levels have the purpose of regulating neutrophil–endothelial interactions, lower levels of NO probably play a role in stimulating the TEM route [53]. In addition, ICAM-1, which is actively involved in TEM, seems to be inversely related to lung function and proportionally related to the severity of emphysema on CT scans [13].
Endothelial apoptosis	Apoptosis is a process regulated by the cell in response to various stimuli or triggers, such as DNA damage or oxidative stress [54]. Initial clinical trials suggested pulmonary vascular endothelial cell apoptosis may plays a significant role in emphysema development [13,54,55].
Endothelial cell senescence	In patients with COPD, especially smokers, oxidative stress is obviously more elevated as a result of many altered mechanisms. Consequently, this results in an accelerated senescence, which is related to BPCO development and to increases in systemic inflammation [56]. Interestingly, it has been proven that corticosteroids appear to have beneficial role in protecting pulmonary endothelial cells from cell senescence [56].
VEGF	VEGF is released in response to hypoxia and plays the role of a growth factor for endothelial cells. VEGF expression prevents endothelial cell apoptosis and induces cell proliferation [13]. Levels of VEGF have been shown to be decreased in patients with COPD [13,28].

**Table 2:** List of the selected articles reporting the patient and study characteristics.

Study	Pop (*n*)	Age (Years)	Comorbidities (%)	Smoking History (%)	Assessment Methods	Aim of the Study
Vaes AW et al. [10]	30	64 ± 7	60% were hypertensive, 40% had (pre)diabetes, 77% had dyslipidaemia, 15% had markedly high levels of PCR.	43.3% were current smokers, 53.3% were ex-smokers.	High-resolution images of ocular fundus.	The primary aim was to assess the effects of exercise on retinal microvasculature in COPD patients.
Vaes AW et al. [12]	246	64.4 ± 8.5	68.3% were hypertensive, 24% had (pre)diabetes, 58.1% had dyslipidaemia, 14.2% had markedly high levels of PCR.	28% were current smokers, 65.9% were ex-smokers.	High-resolution images of ocular fundus.	The primary aim was to assess the relationship between retinal vessel widths and pulmonary function tests, disease outcomes, and cardiovascular risk.
Vaes AW et al. [31]	62	64.4 ± 8.4	62.9% were hypertensive, 24.2% had (pre)diabetes, 53.2% had dyslipidaemia, 9.8% had markedly high levels of PCR.	48.4% were current smokers, 51.6% were ex-smokers	Retinal vessel images were used to assess microvascular health, and skin accumulation of AGEs was assessed by skin AF.	Primary aim was to determine the association between skin AF and microvascular health in COPD patients.
Moro et al. [32]	44	76.7	72.7% were hypertensive, 22.7% had diabetes, and higher prevalence of cardiovascular disease than healthy controls.	29.5% were current smokers, 40.9% were ex-smokers.	Evaluated the forearm blood flow induced by both FMD and NMD.	The primary aim was to evaluate the relationship between severity of bronchial obstruction and endothelial dysfunction.
Vaes AW et al. [33]	40	62.8 ± 7.3	62.5% were hypertensive, 32.5% had (pre)diabetes, 85% had dyslipidaemia, 25% had markedly high levels of PCR.	37.5% were current smokers, 62.5% were ex-smokers.	Microvascular endothelial dysfunction was measured with novel EndoPAT.	The primary aim was to expand the knowledge about the relationship between peripheral endothelial function and COPD patients.
Theodorakopoulou MP et al. [34]	_	_	_	_	Review.	The aim was to define the differences in endothelial dysfunction between COPD patients and control cohort.
Rodriguez-Miguelez P et al. [35]	17	56 ± 2	_	_	Brachial artery FMD was used for the assessment of endothelial function; this exam was combined with arterial tonometry.	The primary aim was to investigate the reproducibility of FMD and arterial stiffness in COPD patients.
Ambrosino et al. [36]	_	_	0–86.7% were hypertensive, 0–43.3% had diabetes, 0–56.7% had dyslipidaemia.	70.5–100% had positive smoking history.	Reviewed the data concerning FMD and NMD in COPD patients.	The aim was to bring together and simplify the role of FMD and NMD as markers of endothelial dysfunction and cardiovascular risk assessment.
Vaes AW et al. [37]	_	_	_	_	Reviewed 10 articles that used 4 different assessment methods: OCT, retinal fundus imaging, retinal oximetry, colour Doppler ultrasonography.	The primary aim was to bring together as much information as possible about retinal vessel imaging and COPD patients.
Harris B et al. [39]	3397	64.5 ± 19	Patients without cardiovascular diseases were recruited; 41.6% were hypertensive, 10.4% had diabetes.	14% were current smokers, 33% were ex-smokers.	Microvascular function was assessed by high-resolution images of ocular fundus, measurement of urine albumin and creatin, and myocardial perfusion evaluation.	The primary aim was to define whether systemic microvascular changes are related to lung function and density.

**Table 3:** Spirometry parameters and main findings.

Articles	FEV1 (% Predicted)	FEV1/FVC Ratio	COPD Severity	Main Findings
Vaes AW et al. [10]	44.6 ± 17.4	36.2 (14.1)	3.3% GOLD I, 33.3% GOLD II, 43.3% GOLD III, 20% GOLD IV	Exercise did not lead to any significant changes in retinal blood vessel diameters. The article suggested that this might be due to inappropriate endothelium vasodilatory response, or inadequate exercise intensity during the test. The study cohort was small in terms of GOLD stage I patients.
Vaes AW et al. [12]	47.3 ± 19.9	35.9 ± 13.0	7.7% GOLD I, 28% GOLD II, 46.7% GOLD III, 17.5% GOLD IV	59% patients showed retinal abnormalities (most common sign was vessel tortuosity). These alterations seem to be related with cardiovascular risk, and above all with hypertension and levels of systemic inflammation. A relationship with lung function parameters was not detected, which is supposedly due to the already highly compromised lung function.
Vaes AW et al. [31]	45.0 ± 20.7	34.8 ± 14.5	8.1% GOLD I, 22.6% GOLD II, 48.4% GOLD III, 21% GOLD IV	Demonstrated an independent association between skin AF and retinal vessel diameter as well as with lower pulmonary functional tests, potentially adding a new tool for the assessment of endothelial function.
Moro et al. [32]	[1.43 L/min]	_	_	The study shows evidence about a predictable inverse relationship between endothelial vasodilatation and bronchial obstruction in COPD. The latter is strictly associated with bronchial inflammation, which in turn is associated with systemic inflammation.
Vaes AW et al. [33]	45.8 ± 17.5	35.8 ± 13.3	7.5% GOLD I, 25% GOLD II, 52.5% GOLD III, 15% GOLD IV	Patients with peripheral endothelial dysfunction demonstrated a lower maximal aerobic capacity, the latter being evaluated by VO2, which is measured using CPET. Nevertheless, using EndoPAT, an association between endothelial function and systemic inflammation was not found.
Theodorakopoulou MP et al. [34]	Values are highly variable depending on the article chosen.			Looking at almost all available functional methods, a significant endothelial impairment was found in COPD patients compared to healthy controls.
Rodriguez-Miguelez P et al. [35]	51.5 ± 3.4	54.8 ± 3.8	23% GOLD I, 53% GOLD II, 24% GOLD III	This study proved for the first time the reproducibility of both FMD in the evaluation of endothelial function and PWV to assess arterial stiffness in COPD patients.
Ambrosino et al. [36]	Vary from 41 to 61.9	Vary from 43 to 59	10% GOLD I, 43.3% GOLD II, 26.7% GOLD III, 20% GOLD IV	It has been shown that patients with a more severe disease, expressed by GOLD classification, had wider gaps in FMD analysis between COPD and healthy controls. Moreover, it has been proven that FMD is strictly associated with endothelial disfunction and cardiovascular risk.
Vaes AW et al. [37]	Values are highly variable depending on the article chosen.			Changes in retinal microvasculature showed higher rates in COPD patients compared to healthy controls.
Harris B et al. [39]	95.7	74.5	_	This article introduced new systemic markers for endothelial damage, all of which are associated with lung function, suggesting that COPD patients show diffuse microvascular alterations.

## Data Availability

Data sharing not applicable.

## Abbreviations

COPD: chronic obstructive pulmonary disease; NVC: nailfold video capillaroscopy; FMD: flow-mediated dilation; FEV1: forced expiratory volume during the first second; TEM: transendothelial migration; NO: nitric oxide; RP: Raynaud’s phenomenon; SRP: secondary Raynaud’s phenomenon; SSc: systemic sclerosis; VEGF: vascular endothelial growth factor; IPF: idiopathic pulmonary fibrosis; NMD: nitrate-mediated dilatation; OCT: optical coherence tomography; FMSF: flow-mediated skin fluorescence; VOP: forearm blood flow after bradykinin infusion; PAT: peripheral arterial tonometry; CPET: cardiopulmonary exercise test; PWV: pulse wave velocity; AF: skin autofluorescence; AGEs: advanced glycation end-products.

## References

[1] Celli B., Fabbri L., Criner G., Martinez F.J., Mannino D., Vogelmeier C., Montes de Oca M., Papi A., Sin D.D., Han M.K. (2022). Definition and Nomenclature of Chronic Obstructive Pulmonary Disease: Time for Its Revision. Am. J. Respir. Crit. Care Med..

[2] Confalonieri M., Braga L., Salton F., Ruaro B., Confalonieri P. (2023). Chronic Obstructive Pulmonary Disease Definition: Is It Time to Incorporate the Concept of Failure of Lung Regeneration?. Am. J. Respir. Crit. Care Med..

[3] Chen W., Thomas J., Sadatsafavi M., FitzGerald J.M. (2015). Risk of cardiovascular comorbidity in patients with chronic obstructive pulmonary disease: A systematic review and meta-analysis. Lancet Respir. Med..

[4] Cavaillès A., Brinchault-Rabin G., Dixmier A., Goupil F., Gut-Gobert C., Marchand-Adam S., Meurice J.C., Morel H., Person-Tacnet C., Leroyer C. (2013). Comorbidities of COPD. Eur. Respir. Rev..

[5] Agustí A., Celli B.R., Criner G.J., Halpin D., Anzueto A., Barnes P., Bourbeau J., Han M.K., Martinez F.J., Montes de Oca M. (2023). Global Initiative for Chronic Obstructive Lung Disease 2023 Report: GOLD Executive Summary. Eur. Respir. J..

[6] Petty T.L. (2006). The history of COPD. Int. J. Chron. Obstruct. Pulmon. Dis..

[7] Forey B.A., Thornton A.J., Lee P.N. (2011). Systematic review with meta-analysis of the epidemiological evidence relating smoking to COPD, chronic bronchitis and emphysema. BMC Pulm. Med..

[8] Bagdonas E., Raudoniute J., Bruzauskaite I., Aldonyte R. (2015). Novel aspects of pathogenesis and regeneration mechanisms in COPD. Int. J. Chron. Obstruct. Pulmon. Dis..

[9] Lim S., Lam D.C., Muttalif A.R., Yunus F., Wongtim S., Lan le T.T., Shetty V., Chu R., Zheng J., Perng D.W. (2015). Impact of chronic obstructive pulmonary disease (COPD) in the Asia-Pacific region: The EPIC Asia population-based survey. Asia Pac. Fam. Med..

[10] Vaes A.W., Spruit M.A., Van Keer K., Barbosa-Breda J., Wouters E.F.M., Franssen F.M.E., Theunis J., De Boever P. (2020). Structural analysis of retinal blood vessels in patients with COPD during a pulmonary rehabilitation program. Sci. Rep..

[11] Lim M., Sasongko M.B., Ikram M.K., Lamoureux E., Wang J.J., Wong T.Y., Cheung C.Y. (2013). Systemic associations of dynamic retinal vessel analysis: A review of current literature. Microcirculation.

[12] Vaes A.W., Spruit M.A., Goswami N., Theunis J., Franssen F.M.E., De Boever P. (2021). Analysis of retinal blood vessel diameters in patients with COPD undergoing a pulmonary rehabilitation program. Microvasc. Res..

[13] Green C.E., Turner A.M. (2017). The role of the endothelium in asthma and chronic obstructive pulmonary disease (COPD). Respir. Res..

[14] Skurikhin E., Pershina O., Zhukova M., Widera D., Pan E., Pakhomova A., Krupin V., Ermakova N., Skurikhina V., Sandrikina L. (2021). Spiperone Stimulates Regeneration in Pulmonary Endothelium Damaged by Cigarette Smoke and Lipopolysaccharide. Int. J. Chron. Obstruct Pulmon Dis..

[15] Karnati S., Seimetz M., Kleefeldt F., Sonawane A., Madhusudhan T., Bachhuka A., Kosanovic D., Weissmann N., Krüger K., Ergün S. (2021). Chronic Obstructive Pulmonary Disease and the Cardiovascular System: Vascular Repair and Regeneration as a Therapeutic Target. Front. Cardiovasc. Med..

[16] Goldenberg N.M., Kuebler W.M. (2015). Endothelial cell regulation of pulmonary vascular tone, inflammation, and coagulation. Compr. Physiol..

[17] Flammer A.J., Anderson T., Celermajer D.S., Creager M.A., Deanfield J., Ganz P., Hamburg N.M., Lüscher T.F., Shechter M., Taddei S. (2012). The assessment of endothelial function: From research into clinical practice. Circulation.

[18] Endemann D.H., Schiffrin E.L. (2004). Endothelial dysfunction. J. Am. Soc. Nephrol..

[19] Peinado V.I., Barbera J.A., Ramirez J., Gomez F.P., Roca J., Jover L., Gimferrer J.M., Rodriguez-Roisin R. (1998). Endothelial dysfunction in pulmonary arteries of patients with mild COPD. Am. J. Physiol..

[20] Barnes P.J., Celli B.R. (2009). Systemic manifestations and comorbidities of COPD. Eur. Respir. J..

[21] Anderson T.J., Gerhard M.D., Meredith I.T., Charbonneau F., Delagrange D.,  Creager M.A., Selwyn A.P., Ganz P. (1995). Systemic nature of endothelial dysfunction in atherosclerosis. Am. J. Cardiol..

[22] Tavakol M.E., Fatemi A., Karbalaie A. (2015). Nailfold Capillaroscopy in Rheumatic Diseases: Which Parameters Should Be Evaluated?. Biomed. Res. Int..

[23] Screm G., Mondini L., Confalonieri P., Salton F., Trotta L., Barbieri M., Mari M., Reccardini N., Della Porta R., Kodric M. (2024). Nailfold Capillaroscopy Analysis Can Add a New Perspective to Biomarker Research in Antineutrophil Cytoplasmic Antibody-Associated Vasculitis. Diagnostics.

[24] Cutolo M., Sulli A., Smith V. (2013). How to perform and interpret capillaroscopy. Best Pract. Res. Clin. Rheumatol..

[25] Bernero E., Sulli A., Ferrari G., Ravera F., Pizzorni C., Ruaro B., Zampogna G., Alessandri E., Cutolo M. (2013). Prospective capillaroscopy-based study on transition from primary to secondary Raynaud’s phenomenon: Preliminary results. Reumatismo.

[26] Bruni C., Frech T., Manetti M., Rossi F.W., Furst D.E., De Paulis A., Rivellese F., Guiducci S., Matucci-Cerinic M., Bellando-Randone S. (2018). Vascular Leaking, a Pivotal and Early Pathogenetic Event in Systemic Sclerosis: Should the door be closed?. Front. Immunol..

[27] Mansueto N., Rotondo C., Corrado A., Cantatore F.P. (2021). Nailfold capillaroscopy: A comprehensive review on common findings and clinical usefulness in non-rheumatic disease. J. Med. Investig..

[28] Guo X., Lin Y., Lin Y., Zhong Y., Yu H., Huang Y., Yang J., Cai Y., Liu F., Li Y. (2022). PM2.5 induces pulmonary microvascular injury in COPD via METTL16-mediated m6A modification. Environ. Pollut..

[29] Corrado A., Carpagnano G.E., Gaudio A., Foschino-Barbaro M.P., Cantatore F.P. (2010). Nailfold capillaroscopic findings in systemic sclerosis related lung fibrosis and in idiopathic lung fibrosis. Jt. Bone Spine.

[30] Heitmar R., Summers R.J. (2012). Assessing vascular function using dynamic retinal diameter measurements: A new insight on the endothelium. Thromb. Haemost..

[31] Vaes A.W., Spruit M.A., Reynaert N.L., Franssen F.M.E., Wouters E.F.M., Theunis J., De Boever P. (2020). Skin auto-fluorescence as a measure of advanced glycation end-products is associated with microvascular health in patients with COPD. Microvasc. Res..

[32] Moro L., Pedone C., Scarlata S., Malafarina V., Fimognari F., Antonelli-Incalzi R. (2008). Endothelial dysfunction in chronic obstructive pulmonary disease. Angiology.

[33] Vaes A.W., Spruit M.A., Theunis J., Wouters E.F.M., De Boever P. (2018). Peripheral endothelial function is positively associated with maximal aerobic capacity in patients with chronic obstructive pulmonary disease. Respir. Med..

[34] Theodorakopoulou M.P., Alexandrou M.E., Bakaloudi D.R., Pitsiou G., Stanopoulos I., Kontakiotis T., Boutou A.K. (2021). Endothelial dysfunction in COPD: A systematic review and meta-analysis of studies using different functional assessment methods. ERJ Open Res..

[35] Rodriguez-Miguelez P., Seigler N., Bass L., Dillard T.A., Harris R.A. (2015). Assessments of endothelial function and arterial stiffness are reproducible in patients with COPD. Int. J. Chron. Obstruct Pulmon Dis..

[36] Ambrosino P., Lupoli R., Iervolino S., De Felice A., Pappone N., Storino A., Di Minno M.N.D. (2017). Clinical assessment of endothelial function in patients with chronic obstructive pulmonary disease: A systematic review with meta-analysis. Intern. Emerg. Med..

[37] Vaes A.W., Spruit M.A., Theunis J., Goswami N., Vanfleteren L.E., Franssen F.M.E., Wouters E.F.M., De Boever P. (2018). Looking into the eye of patients with chronic obstructive pulmonary disease: An opportunity for better microvascular profiling of these complex patients. Acta Ophthalmol..

[38] Laisure M., Covill N., Ostroff M.L., Ostroff J.L. (2021). Summarizing the 2021 Updated GOLD Guidelines for COPD. US Pharm..

[39] Harris B., Klein R., Jerosch-Herold M., Hoffman E.A., Ahmed F.S., Jacobs D.R., Klein B.E., Wong T.Y., Lima J.A., Cotch M.F. (2012). The association of systemic microvascular changes with lung function and lung density: A cross-sectional study. PLoS ONE.

[40] Taher R., Sara J.D., Toya T., Shepherd R., Moder K., Lerman L.O., Lerman A. (2020). Secondary Raynaud’s phenomenon is associated with microvascular peripheral endothelial dysfunction. Microvasc. Res..

[41] Rollando D., Bezante G.P., Sulli A., Balbi M., Panico N., Pizzorni C., Negrini S., Brunelli C., Barsotti A., Cutolo M. (2010). Brachial artery endothelial-dependent flow-mediated dilation identifies early-stage endothelial dysfunction in systemic sclerosis and correlates with nailfold microvascular impairment. J. Rheumatol..

[42] Silva I., Teixeira A., Oliveira J., Almeida I., Almeida R., Águas A., Vasconcelos C. (2015). Endothelial Dysfunction and Nailfold Videocapillaroscopy Pattern as Predictors of Digital Ulcers in Systemic Sclerosis: A Cohort Study and Review of the Literature. Clin. Rev. Allerg. Immunol..

[43] Silva I., Loureiro T., Teixeira A., Almeida I., Mansilha A., Vasconcelos C., Almeida R. (2015). Digital ulcers in systemic sclerosis: Role of flow-mediated dilatation and capillaroscopy as risk assessment tools. Eur. J. Dermatol..

[44] Le J.H., Cho K.I. (2014). Association between endothelial function and microvascular changes in patients with secondary Raynaud’s phenomenon. Clin Rheumatol..

[45] Corrado A., Mansueto N., Correale M., Rella V., Tricarico L., Altomare A., Brunetti N.D., Cantatore F.P., Rotondo C. (2024). Flow Mediated Dilation in Systemic Sclerosis: Association with clinical findings, capillaroscopic patterns and endothelial circulating markers. Vascul. Pharmacol..

[46] Ushiyama O., Ushiyama K., Yamada T., Koarada S., Tada Y., Suzuki N., Ohta A., Nagasawa K. (2003). Retinal findings in systemic sclerosis: A comparison with nailfold capillaroscopic patterns. Ann. Rheum. Dis..

[47] Shenavandeh S., Afarid M., Hasanaghaei T., Nazarinia M.A. (2021). Prevalence of retinal changes in patients with systemic sclerosis: The association between retinal vascular changes and nailfold capillaroscopic findings. Reumatologia.

[48] Jakhar D., Grover C., Singal A., Das G.K. (2020). Nailfold Capillaroscopy and Retinal Findings in Patients with Systemic Sclerosis: Is There an Association?. Indian Dermatol. Online J..

[49] Cutolo M., Vanhaecke A., Ruaro B., Deschepper E., Ickinger C., Melsens K., Piette Y., Trombetta A.C., De Keyser F., Smith V. (2018). Is laser speckle contrast analysis (LASCA) the new kid on the block in systemic sclerosis? A systematic literature review and pilot study to evaluate reliability of LASCA to measure peripheral blood perfusion in scleroderma patients. Autoimmun. Rev..

[50] Cutolo M., Trombetta A.C., Melsens K., Pizzorni C., Sulli A., Ruaro B., Paolino S., Deschepper E., Smith V. (2018). Automated assessment of absolute nailfold capillary number on videocapillaroscopic images: Proof of principle and validation in systemic sclerosis. Microcirculation.

[51] Mondini L., Confalonieri P., Pozzan R., Ruggero L., Trotta L., Lerda S., Hughes M., Bellan M., Confalonieri M., Ruaro B. (2023). Microvascular Alteration in COVID-19 Documented by Nailfold Capillaroscopy. Diagnostics.

[52] Gane J., Stockley R. (2012). Mechanisms of neutrophil transmigration across the vascular endothelium in COPD. Thorax.

[53] Burns A.R., Zheng Z., Soubra S.H., Chen J., Rumbaut R.E. (2007). Transendothelial flow inhibits neutrophil transmigration through a nitric oxide-dependent mechanism: Potential role for cleft shear stress. Am. J. Physiol. Heart Circ. Physiol..

[54] Song Q., Chen P., Liu X.-M. (2021). The role of cigarette smoke-induced pulmonary vascular endothelial cell apoptosis in COPD. Respir. Res..

[55] Salton F., Ruaro B., Confalonieri P., Confalonieri M. (2020). Epithelial-Mesenchymal Transition: A Major Pathogenic Driver in Idiopathic Pulmonary Fibrosis?. Medicina.

[56] Paschalaki K., Rossios C., Pericleous C., MacLeod M., Rothery S., Donaldson G.C., A Wedzicha J., Gorgoulis V., Randi A.M., Barnes P.J. (2022). Inhaled corticosteroids reduce senescence in endothelial progenitor cells from patients with COPD. Thorax.

[57] Maricić L., Vceva A., Visević R., Vcev A., Milić M., Serić V., Fijacko V. (2013). Assessment of endothelial dysfunction by measuring von Willebrand factor and exhaled nitric oxide in patients with chronic obstructive pulmonary disease. Coll. Antropol..

[58] A Hatoum O., Binion D.G., Otterson M.F., Gutterman D.D. (2003). Acquired microvascular dysfunction in inflammatory bowel disease: Loss of nitric oxide-mediated vasodilation. Gastroenterology.

